# SCMHBP: prediction and analysis of heme binding proteins using propensity scores of dipeptides

**DOI:** 10.1186/1471-2105-15-S16-S4

**Published:** 2014-12-08

**Authors:** Yi-Fan Liou, Phasit Charoenkwan, Yerukala Sathipati Srinivasulu, Tamara Vasylenko, Shih-Chung Lai, Hua-Chin Lee, Yi-Hsiung Chen, Hui-Ling Huang, Shinn-Ying Ho

**Affiliations:** 1 Institute of Bioinformatics and Systems Biology, National Chiao Tung University, Hsinchu, Taiwan; 2 Department of Biological Science and Technology, National Chiao Tung University, Hsinchu, Taiwan

## Abstract

**Background:**

Heme binding proteins (HBPs) are metalloproteins that contain a heme ligand (an iron-porphyrin complex) as the prosthetic group. Several computational methods have been proposed to predict heme binding residues and thereby to understand the interactions between heme and its host proteins. However, few *in silico *methods for identifying HBPs have been proposed.

**Results:**

This work proposes a scoring card method (SCM) based method (named SCMHBP) for predicting and analyzing HBPs from sequences. A balanced dataset of 747 HBPs (selected using a Gene Ontology term GO:0020037) and 747 non-HBPs (selected from 91,414 putative non-HBPs) with an identity of 25% was firstly established. Consequently, a set of scores that quantified the propensity of amino acids and dipeptides to be HBPs is estimated using SCM to maximize the predictive accuracy of SCMHBP. Finally, the informative physicochemical properties of 20 amino acids are identified by utilizing the estimated propensity scores to be used to categorize HBPs. The training and mean test accuracies of SCMHBP applied to three independent test datasets are 85.90% and 71.57%, respectively. SCMHBP performs well relative to comparison with such methods as support vector machine (SVM), decision tree J48, and Bayes classifiers. The putative non-HBPs with high sequence propensity scores are potential HBPs, which can be further validated by experimental confirmation. The propensity scores of individual amino acids and dipeptides are examined to elucidate the interactions between heme and its host proteins. The following characteristics of HBPs are derived from the propensity scores: 1) aromatic side chains are important to the effectiveness of specific HBP functions; 2) a hydrophobic environment is important in the interaction between heme and binding sites; and 3) the whole HBP has low flexibility whereas the heme binding residues are relatively flexible.

**Conclusions:**

SCMHBP yields knowledge that improves our understanding of HBPs rather than merely improves the prediction accuracy in predicting HBPs.

## Background

Heme binding proteins (HBPs) are metalloproteins that contain a heme ligand (an iron-porphyrin complex) as a prosthetic group. HBPs exist in various forms. The most common hemes are b- and c-types [1]. The b-type heme binds to proteins non-covalently, whereas the vinyl groups of the c-type heme forms covalent bonds with two specific cysteine residues of the Cys-Xaa-Xaa-Cys-His motif [1,2]. Other important hemes include a-, o-, and d-type hemes, which are found in bacteria and eukaryotes [2]. The heme binds to HBPs according to its types. Understanding of the structure-function relationships of heme iron complex or dissociation is useful for rational HBP engineering.

HBPs can perform a variety of biological functions, such as electron transfer [3], diatomic gas transportation/storage [4], chemical catalysis [5], transcriptional regulation [6], ion channel chemosensing [6], circadian clock control [7], and microRNA processing [8]. The functional diversity of HBPs causes them have various special applications, such as in biopolymer films that are used in research into electrocatalysis [9,10] and components of new biocatalysts [11]. The heme enzyme chlorite dismutase has been successfully utilized in studying O_2_-utilizing enzymes [12]. Studies of HBPs and the heme biosynthesis pathway are crucial to the development of efficient cancer phototherapies [13]. Identifying novel HBPs increases their range of applications.

Identifying a novel HBP requires some complex experiments and analysis, such as analysis of its crystal structure and sequence, and biochemical assays [14]. Merkley *et al*. [15] combined histidine affinity chromatography with a proteomics database to improve the identification of c-type heme peptides in liquid chromatography-tandem mass spectrometry experiments. Babusiak *et al*. [16] combined native chromatography with native electrophoresis to examine HBP complexes in murine erythroleukemia cells. Designing artificial HBPs is a good approach to exploring novel applications. Accordingly, understanding the physicochemical properties (PCPs) of HBPs is also crucial. With respect to PCP analysis, previous investigations have examined the isoelectric point, acidity, chemical sensing, oxidation, reduction, and ligand binding catalysis [17] of HBPs. The PCPs and the transportation of electrons have been observed from flavocytochromes [18].

The function diversity of HBPs suggests that the sequences and structures of HBPs are highly variable, limiting homological search methods for their discovery. Most *in silico *investigations focus on heme binding sites and heme binding mechanisms [19-21], rather than the identification of HBPs. Schneider *et al*. [19] examined b-type heme proteins with various folding topologies and elucidated their ability to bind chemically identical heme ligands. They found that the residues from these different topologies cluster at particular interaction "hot spots", and define some common structural heme-binding motifs. Smith *et al*. [20] comprehensively analyzed a dataset of non-homologous HBPs; their results revealed some typical characteristics of the heme groups and their binding sites in proteins with various functionalities. Li *et al*. [21] elucidated the differences between the apo and holo structures of HBPs and found that HBPs generally undergo small conformational changes following heme binding.

As well as being useful in investigating binding sites, several support vector machine (SVM) based methods predict heme binding residues to elucidate the mechanism of heme-protein interactions. HemeBIND [22] is an SVM-based ensemble predictor that uses two feature sets position specific scoring matrices (PSSM) and structural information (comprising solvent accessibility, depth, and a protrusion index). HemeNET [23] uses topological properties of binding residues from the 3D interaction networks to improve its predictive performance. Xiong *et al*. [24] and TargetS [25] proposed sequence-based prediction methods that took into account the low availability of the 3D structures of HBPs.

Since the aforementioned experimental approaches are time-consuming and labor-intensive, and few effective *in silico *methods for identifying HBPs are available, this work proposes a novel method (SCMHBP) for predicting and analyzing HBPs from primary sequences. SCMHBP uses a newly-developed scoring card method (SCM) that was proposed by Huang *et al*. [26-28] for estimating scores of the propensity of amino acid and dipeptides to be HBPs. These propensity scores of dipeptides are calculated using the difference between the dipeptide compositions of HBPs and non-HBPs and are further optimized by applying an intelligent genetics algorithm [29]. Consequently, the propensity scores of amino acids can be utilized to discover highly related PCPs of HBPs by exploring 531 PCPs in the AAindex database [30]. The advantages of SCMHBP are its accurate predictions, simple methodology, and high interpretability.

Two presently available datasets of HBPs are HemeBind145 [22] and TargetS233 [25]. However, no dataset of non-HBPs is available. Therefore, first, a new dataset is established from the SwissPort dataset consisting of 747 HBPs (selected using a Gene Ontology term, GO:0020037) and 747 non-HBPs (selected from 91,414 putative non-HBPs) with an identity of 25%. This dataset is divided into both training and test datasets. The TargetS233 dataset is enlarged by retrieving HBPs from the new version of BioLiP, which is a ligand-protein binding database [31]. The recollected dataset of 311 HBPs is named TargetS311. The training and mean test accuracies of SCMHBP when applied to three independent test datasets are 85.90% and 71.57%, respectively. SCMHBP is better than some typical methods such as SVM, decision tree J48, and Bayes classifier-based methods, which are implemented using WEKA [32].

To characterize HBPs, the propensity scores of individual amino acids and dipeptides are investigated to elucidate the interactions between heme and its host proteins. Additionally, based on the correlation coefficient (*R *value) between the propensity scores of amino acids and the PCPs in AAindex, three informative PCPs are identified; they are 1) SNEP660103 with *R *= 0.604 described as ''principal component III'' [33], 2) TAKK010101 with *R *= 0.576 described as ''side-chain contribution to protein stability'' [34], and 3) KARP850101 with *R *= -0.555 described as ''flexibility parameter for no rigid neighbors'' [35]. Further analyzing the PCPs reveals that 1) high aromaticity affects the functionality of HBP, suggesting that conserved residues with aromatic side chains importantly affect the performance of specific HBP functions, 2) a hydrophobic environment plays an important role in the interaction between heme and binding sites, and 3) the HBP has a low overall flexibility whereas the heme binding residues are relatively flexible. The high side-chain contribution to the stability of HBPs arises from those aromatic and non-polar residues, residues' ability to form non-covalent bonds.

## Materials and methods

**Figure 1:**
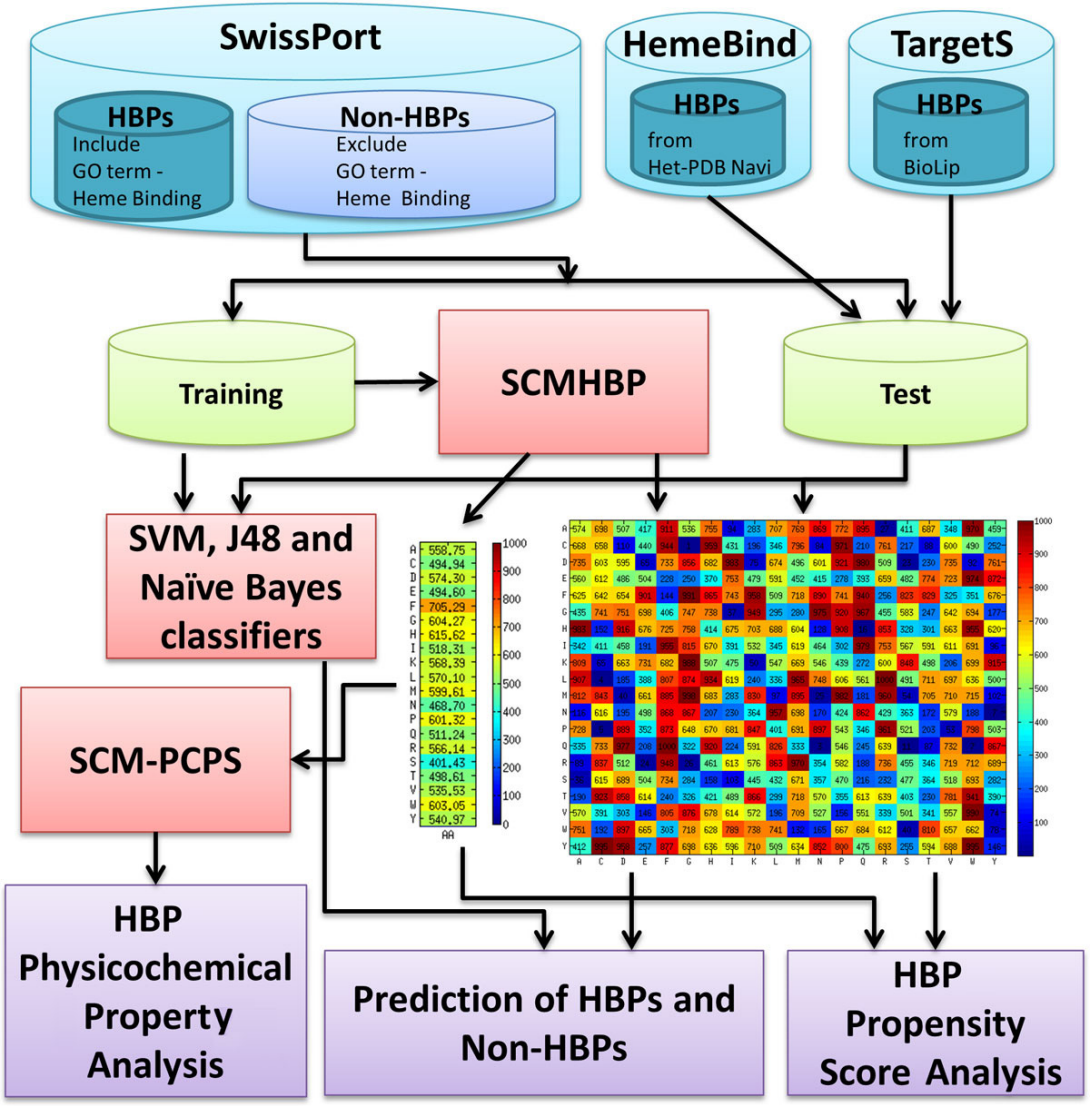
**Flowchart of the system design for prediction and analysis of heme binding proteins (HBPs)**.

This work proposes the SCMHBP method, which is based on a scoring card method (SCM), to estimate the propensity of 400 dipeptides and 20 amino acids for predicting and analysis HBPs from protein sequences. Figure 1 presents the flowchart of the design of systems to predict and analyze HBPs, which involve datasets, methods, and the analysis of propensity scores.

### Datasets

Two datasets of HBPs without non-HBPs, HemeBind145 [22] and TargetS311 [25], are available. To design predictors of HBPs, a non-redundant dataset of 747 HBPs that are selected using a Gene Ontology term GO:0020037 and 747 non-HBPs that are selected from 91,414 putative non-HBPs (filtering 395 sequences with a non-amino acid character from 92,309 putative non-HBPs) is established. The dataset is divided into two sets, HBPGO-TRN1000 and HBPGO-TST494 for training and testing, respectively. Table 1 summarizes the used training dataset of HBPGO-TRN1000 for designing HBP predictors and three test datasets for evaluating these predictors.

**Table 1:** The training dataset HBPGO-TRN1000 for designing HBP predictors and three test datasets for evaluating the predictors.

Dataset	Sequence Identity (%)	HBP Number	Non-HBP Number	Total Number
HBPGO-TRN1000	25	500	500	1000
HBPGO-TST494	25	247	247	494
HemeBind145 [22]	30	145	0	145
TargetS311 [25]	40	311	0	311

#### HBPGO-TRN1000 and HBPGO-TST494

The datasets are established by collecting sequences from the SwissProt database (version:2013_09) [36] using the GO term GO:0020037 to define HBPs from the GO database [37]. If the sequence is annotated using this GO term, then it is regarded as an HBP; otherwise, it is a putative non-HBP. The sequence identity of any pair of a sequences is reduced to 25% using USEARCH [38]. Hence, 747 HBPs and 91,414 putative non-HBPs are obtained. The 747 HBPs are divided into two sets 500 HBPs for training and 247 HBPs for testing.

Owing to the large number of putative non-HBPs, the identification of a small set of non-HBPs for designing predictors is an issue. Ten candidate datasets of 500 non-HBPs were prepared (to establish a balanced dataset) by random selection from the 91,414 non-HBPs. Table 2 presents the prediction performances of the score cards that were generated from these ten randomly selected negative datasets. The mean accuracy of these ten score cards is 85.22%. The best of ten datasets that yields the highest training accuracy is used as the final dataset of non-HBPs in HBPGO-TRN1000. This best training accuracy is 86.60%, with a sensitivity of 0.86 and a specificity of 0.87.

**Table 2:** The performance of 10 randomly selected negative datasets

Number	Training (%)	MCC	Sensitivity	Specificity
1	86.60	0.47	0.68	0.79
2	84.90	0.43	0.66	0.77
3	85.30	0.40	0.67	0.73
4	85.00	0.41	0.73	0.68
5	86.00	0.41	0.67	0.64
6	85.20	0.41	0.71	0.70
7	84.40	0.43	0.70	0.73
8	84.90	0.41	0.76	0.65
9	86.00	0.42	0.75	0.67
10	83.90	0.44	0.70	0.73

average	85.22	0.42	0.70	0.71

In HBPGO-TST494, the 247 sequences of are randomly selected from 90,914 non-HBPs that are not in the negative part of HBPGO-TRN1000. We note that these sequences does not involve in the training process.

#### HemeBind145

Two datasets [22] to predict HBP binding sites: 1) one dataset comprised 72 non-redundant HBPs that were collected from the Het-PDB Navi. database (version at May 2010) [39] and 2) the other dataset comprised 75 non-redundant HBPs was presented by Fufezan *et al*. [2]. In these datasets, pair of chains has a sequence identity of more than 30%. The two datasets are combined by removing two sequences with a non-amino acid character to generate the new dataset, HemeBind145, of size 145.

#### TargetS311

In the TargetS method [25], 233 HBPs that were collected from the ligand-protein binding database BioLiP [31] with a sequence identity of 40% were used. BioLiP is a semi-manually curated database for high-quality, biologically relevant ligand-protein binding interactions. In this work, 311 HBPs with a sequence identity of 40% were collected from the new version of BioLiP as the independent test dataset, referred to as TargetS311.

## Methods

### Typical classification methods

To the best our knowledge, few effective methods or tools for predicting HBPs from sequences have been proposed. To develop an accurate predictor, some typical classification methods such as those based on the SVM, J48, and the used of Bayes classifiers with a single type of sequence features have been implemented for performance comparison. The SVM is widely recognized to be an accurate classifier for prediction proteins with a specific function. Generally, the predictive performance of an SVM with effective features is regarded as the gold standard for evaluating predictors. Radial basis SVM classifiers are implemented using the LIBSVM package [40]. The SVM parameters are evaluated by using a grid search method to maximize the ten folds cross validation (10-CV) accuracy on the training dataset. Some commonly used features such as amino acid composition (AAC), dipeptide composition (DPC), normalized PSSM (PSSM400) [41], and 531 PCPs in the AAindex are evaluated in the design of predictors.

The J48 and Bayes classifiers are implemented using WEKA [32]. J48 refers to the decision tree classifier that is generated by the C4.5 algorithm that was developed by Quinlan [42]. The Naïve Bayes classifier is a statistical classifier that can predict the probability of class membership under the assumption of the mutual independence of features [43].

### Scoring card method

The scoring card method (SCM) is a newly-published classification method for predicting proteins with a particular function and providing insight into the characteristics of proteins in primary sequences. Huang *et al*. developed this method [26-28]. Unlike the complex classification mechanism of the SVM, which is not easily understood by biologists, the used of SCM to estimate the propensities of amino acids and dipeptides to be the function of interest is a simple and easily interpretable method of prediction and analysis. A comparison of prediction accuracies, the SCM is slightly worse than, or comparable to, the SVM when used with dipeptide features [26-28]. The advantages of SCM are three folds. First, the classification mechanism of the SCM adopts a weighted sum of composition and propensity scores of dipeptides to score the queried protein. Compared with the hyperplane of the SVM, a threshold value in the SCM is used to classify proteins, and this value is easily understood and manipulated by biologists. Secondly, the propensity scores of dipeptides and amino acids can be utilized to identify the PCPs that will provide information about a global property of general proteins in a further analysis of the proteins' characteristics. Thirdly, the SCM is a general-purpose method for identifying protein sequences with a particular function. The proposed SCMHBP method is based on the SCM that is applied to the training dataset of HBPGO-TRN1000. For completeness, the used SCM and the SCMHBP algorithm are described below.

The main procedure in the design SCM-based predictors can be conducted without modification, and consists of the following steps; 1) preparing both positive and negative sequences in a training dataset as inputs (500 HBPs and 500 non-HBPs in HBPGO-TRN1000 in this study); 2) generating an initial scoring card with 400 propensity scores of dipeptides using a simple statistical method; 3) obtaining propensity scores of 20 amino acids from those of 400 dipeptides; 4) optimizing the initial scoring card using a global optimization method, and 5) generating a binary SCM classifier with a threshold value as an output of the procedure. More details of the SCM method and its applications can be found elsewhere [26-28]. The algorithm of SCMHBP is as follows.

Step 1: Prepare a training dataset HBPGO-TRN1000 that comprises 500 HBPs and 500 non-HBPs.

Step 2: Generate an initial scoring card (SCMInit) that consists of 400 propensity scores of dipeptides, obtained by subtracting the dipeptides contents in non-HBPs from those in HBPs. Then, the scores of all dipeptides are normalized into the range [0, 1000].

Step 3: Calculate the propensity score of each amino acid × by averaging 40 propensity scores of dipeptides that contain X.

Step 4: Optimize the scoring card (Scard) of dipeptides using an intelligent genetic algorithm, IGA [29]. The fitness function of IGA is to maximize both the prediction accuracy in terms of the area under the ROC curve (AUC) [44] and the Pearson's correlation coefficient (R value) between the initial and optimized propensity scores of 20 amino acids. To prevent overfitting, the fitness function is calculated by performing a 10-CV assessment, and is as follows (*W*_1_ = 0.9 and *W*_2 _= 0.1 in this study).

(1) $$Max\;Fit\left(Scard\right)=W_{1}\times AUC+W_{2}\times R$$

Step 5: Classify a query sequence *P *based on the scoring function *S(P) *and determine a threshold value that yields the highest training accuracy. The variables $w_{i}$ and $S_{i}$ are the content and propensity score of the *i*-th dipeptide, respectively. *P *is classified as an HBP when *S(P) *exceeds the threshold value; otherwise, *P *is a non-HBP.

(2) $$S\left(P\right)=\overset{400}{\underset{i=1}{\sum }}w_{i}S_{i}.$$

### Identifying informative physicochemical properties

The physicochemical properties of amino acids are widely recognized as effective features for predicting and analyzing the functions of proteins from primary sequences [26-28]. The AAindex database consists 544 indices that were extracted from the published literatures and represent various physicochemical and biological properties of amino acids [30]. Each physicochemical property of amino acids is specified by a set of 20 numerical values. The property with the value 'NA' in a value set of amino acid index is removed. Finally, 531 properties are utilized in the following analysis. The propensity scores of amino acids, estimated by SCMHBP, facilitate the discovery of informative PCPs, providing insight into the characteristics of HBPs. The method of identification of informative PCPs, based on the SCM (SCM-PCPs), is composed of two main steps.

The first step is to compute the R values between the propensity scores of the amino acids and all 531 PCPs in AAindex. A large value of *R *suggests that the physicochemical or biological property is highly correlated with property of HBPs. The property with an absolute value of *R *> 0.5 is preferred as a candidate for subsequent analysis.

The second step is to identify the informative PCPs from all candidates, based on domain knowledge of the function of the investigated proteins (HBPs in this work). Generally, the PCPs of amino acids in AAindex are obtained under a particular condition or for a specific family of proteins that is not satisfied are not suited to analysis.

The compositions of the amino acids in HBPs and non-HBPs effectively represent a property of HBPs. Accordingly, the composition property can also be used like the propensity scores of amino acids in using SCM-PCPs [28]. The identified PCPs offer a clue to the characteristics of HBPs.

## Results and discussion

### Propensity scores of amino acids and dipeptides

**Figure 2:**
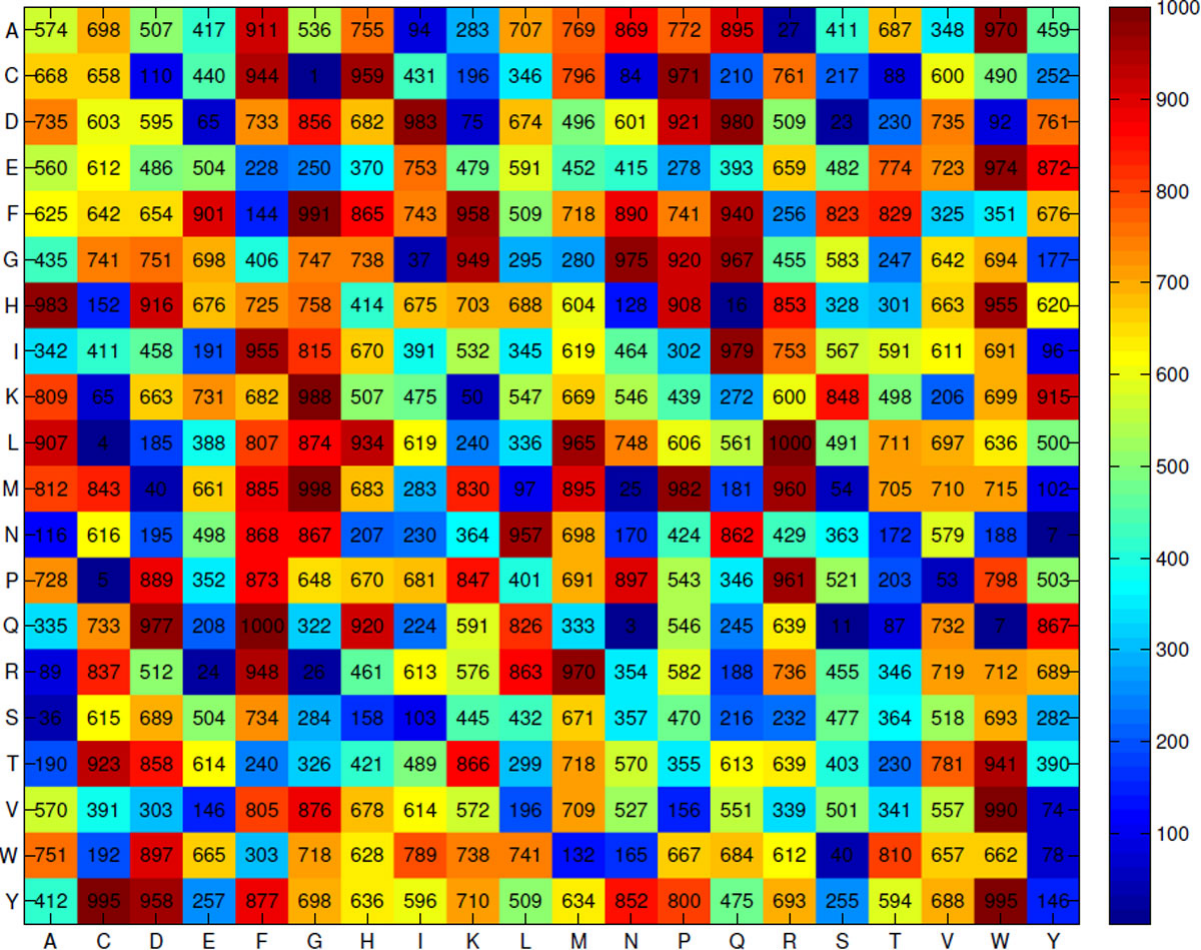
**Heat map of the heme protein propensity scores of dipeptides**.

Figure 2 presents the propensities of 400 dipeptides to be HBPs, obtained by SCMHBP using the training dataset HBPGO-TRN1000 consisting of 500 HBPs and 500 non-HBPs. Table 3 presents the propensity scores of 20 amino acids that were derived from the propensity scores of 400 dipeptides. The table also presents the amino acid compositions of HBPs and non-HBPs with 199,772 and 200,188 residues, respectively. The correlation coefficient *R *between the propensity scores of the amino acids and difference between the amino acids composition of HBPs and non-HBPs is 0.92. This high correlation coefficient reveals that the propensity scores of amino acids can be used effectively to distinguish HBPs from non-HBPs. The *R *= -0.05 between the propensity scores of the amino acids and composition of HBPs suggests that the propensity scores reflect the difference between the composition in HBPs and non-HBPs, rather than the composition in HBPs only.

**Table 3:** The propensity scores of amino acids to be a heme binding protein (HBP) and amino acid composition (%).

Amino acid	Heme protein Score (Rank)	Composition of HBP: A (%)	Composition of Non-HBP: B (%)	Composition difference: A-B (%)
F-Phe	705.3(1)	5.08	4.07	1.01
H-His	615.6(2)	2.59	2.26	0.33
G-Gly	604.3(3)	6.66	6.00	0.66
W-Trp	603.1(4)	1.47	1.08	0.39
P-Pro	601.3(5)	5.17	4.70	0.47
M-Met	599.6(6)	2.69	2.17	0.52
D-Asp	574.3(7)	5.41	5.33	0.08
L-Leu	570.1(8)	10.28	9.77	0.51
K-Lys	568.3(9)	5.80	6.19	-0.39
R-Arg	566.1(10)	5.56	5.12	0.44
A-Ala	558.8(11)	7.59	7.21	0.38
Y-Tyr	541.0(12)	3.04	3.17	-0.13
V-Val	535.5(13)	6.41	6.29	0.12
I-Ile	518.3(14)	5.77	6.16	-0.39
Q-Gln	511.2(15)	3.61	4.10	-0.49
T-Thr	498.6(16)	5.31	5.63	-0.32
C-Cys	494.9(17)	1.40	1.63	-0.23
E-Glu	494.6(18)	5.95	6.49	-0.54
N-Asn	468.7(19)	4.01	4.89	-0.88
S-Ser	401.4(20)	6.21	7.73	-1.52

R	1.00	-0.05	-0.30	0.92

The total number of amino acids in the used datasets of HBPs and non-HBPs is 199,772 and 200,188, respectively.

**Figure 3:**
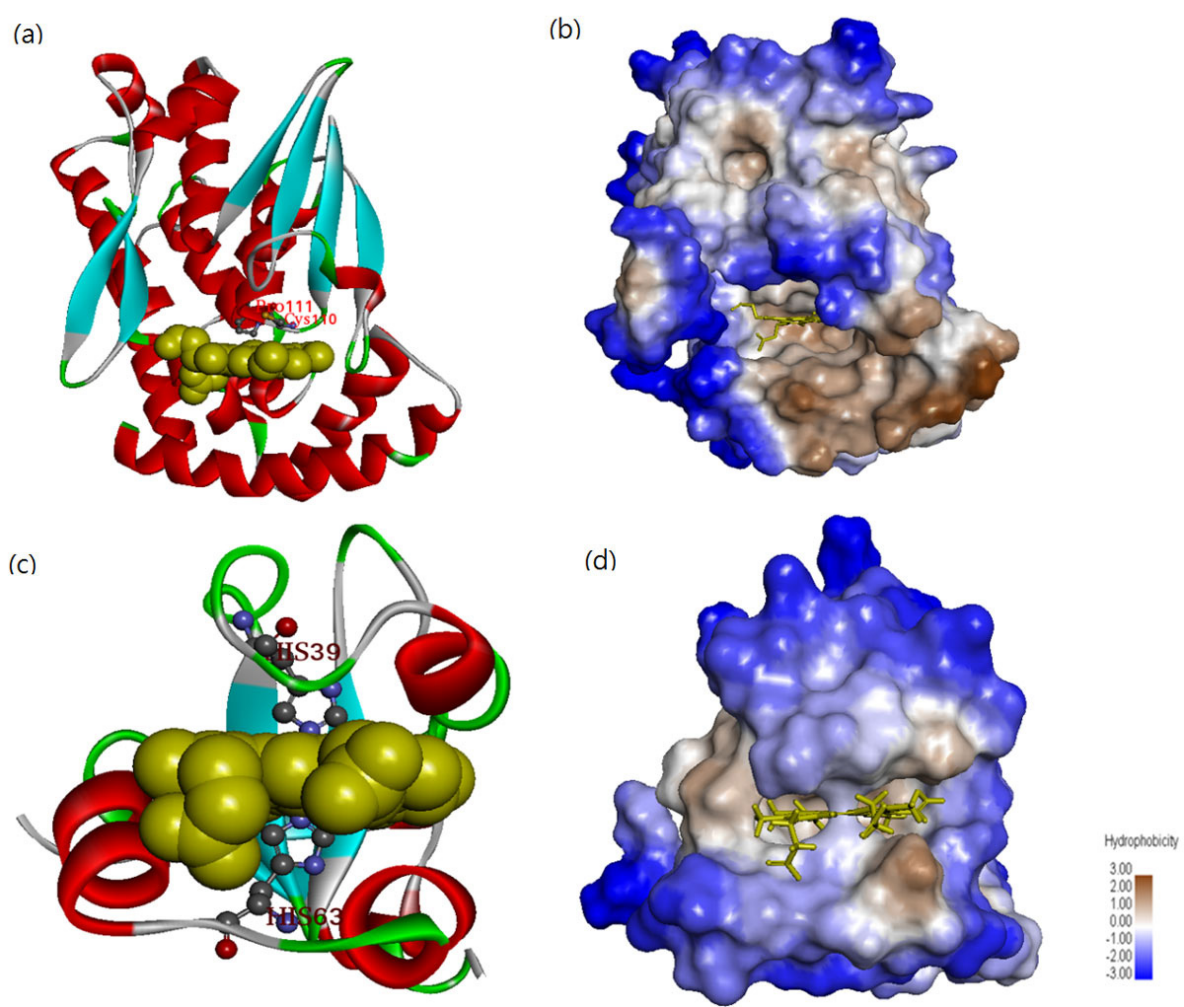
**The secondary structures and surface hydrophobicity of 2PBJ and 1CYO**. The heme is represented as spheres. The color of the surfaces represents the level of hydrophobicity. The blue, white, and brown colors represent low, mediate, and high hydrophobicity, respectively. (a) The structures of 2PBJ where a CP motif is located near the heme for the interaction with the heme. (b) Surface hydrophobicity of 2PBJ. The heme is represented as yellow sticks. (c) The structures of 1CYO. (d) Surface hydrophobicity of 1CYO. Protein structures are drawn using Discovery studio 4.0.

The c-type heme vinyl group forms covalent bonds with two particular cysteine residues of the Cys-Xaa-Xaa-Cys-His motif. The heme c proteins with histidine as an axial ligand have the classic CXXCH heme c binding motif. The heme b proteins have cysteine as an axial ligand [21]. The propensity score of CH (Cys-His) is as high as 959 (Figure. 2), which is consistent with the fact that CH is a dipeptide motif of HBPs. Li *et al*. [21] analyzed the structures of 125 heme-binding proteins chains and found that the dipeptide CP (Cys-Pro) heme regulatory motifs have an important structural role in protein-heme interactions when Cys functions as an axial ligand with heme iron. Ogawa *et al*. [45] indicated that four CP motifs are important to both the Bach1-heme interaction and the heme-mediated inhibition of DNA binding. Zenke-Kawasaki *et al*. [46] found that CP motifs have a critical role in the heme-Bach1 interaction, which regulates the expression of the heme oxygenase-1 gene. The propensity score of the dipeptide CP is as high as 971, suggesting that the dipeptide motif of HBPs has a high propensity score. Restated, the dipeptides with high scores have the potential to be dipeptide motifs. To elucidate the interaction between the CP motif and heme, 2PBJ is chosen from the HBP dataset [23]. Figure 3(a) presents the secondary structure of 2PBJ when a CP motif is a perpendicular to the heme plane. The CP motif is located closed to the heme and interacts with it.

The five amino acids with the highest propensity scores are Phe, His, Gly, Trp, and Pro, whereas the residues with the lowest scores are Ser, Asn, Glu, Cys, and Thr. Smith *et al*. [20] found that His predominates among the other amino acids as an axial ligand to heme iron. The relative frequency of His in the HBPs decreases to the background level when the axial ligands are removed [23]. Smith et al. [20] noted the important role of Trp and Phe and Tyr in protein-heme interactions. Liu and Hu measured the relative importance of the amino acids in heme binding interfaces and suggested that His, Phe, and Trp are overrepresented in binding pockets [22]. Notably, the aromatic and non-polar residue Phe has the highest score and can form non-covalent bonds.

### Performance of HBP predictors

The training dataset HBPGO-TRN1000 was used to design SCMHBP and compared methods, SVM, J48, and Bayes classifiers. For each classifier, four types of features were evaluated they were 1) amino acid composition (AAC), 2) dipeptide composition (DPC), 3) normalized PSSM (PSSM400), and 4) 531 PCPs in AAindex. Table 4 compares of prediction accuracies of SCMHBP and the compared methods.

**Table 4:** The comparisons of prediction accuracies (%) between SCMHBP and some methods.

Method	HBPGO-TRN1000	HBPGO-TST494	HemeBind145	TargetS311	Mean test
SCMHBP*	85.90 ± 0.42	72.79 ± 1.21	67.24 ± 2.89	74.69 ± 2.48	71.57 ± 7.83
SVM-AAC	91.00	64.57	66.21	75.56	68.78
SVM-DPC	97.70	78.54	63.45	74.60	72.20
SVM-PSSM400	91.00	87.45	66.21	74.28	75.98
SVM-AAindex	87.60	76.52	71.72	80.39	76.21
J48-AAC	93.80	66.80	65.52	67.20	66.51
J48-DPC	97.90	64.78	60.00	68.81	64.53
J48-PSSM400	97.70	75.30	61.38	62.38	66.35
J48-AAindex	98.60	68.22	62.76	70.42	67.13
Bayes-AAC	69.20	67.40	71.03	62.70	67.04
Bayes-DPC	67.40	63.77	73.79	71.38	69.65
Bayes-PSSM400	57.80	55.30	85.52	82.64	74.49
Bayes-AAindex	63.60	63.20	61.38	55.95	60.18

Mean accuracy	84.55	69.63	69.02	72.00	70.22

* The results are the averages of the performances with 10 difference score cards from 10 independent runs.

The training accuracy of SCMInit and the mean accuracy of SCMHBP are 71.30% and 85.90%, respectively. The optimization of the scoring card by using an intelligent genetic algorithm (IGA) [29] increases the training accuracy to 14.60%. The SCMHBP method uses the 10-CV assessment to prevent overtraining in training the model. This optimization method seeks to maximize the area under the ROC curve (AUC) [44] to generate balanced sensitivity and specificity. The mean test accuracies of SCMHBP that are obtained using the DPC features on the three test datasets HBPGO-TST494, HemeBind145, and TargetS311, are 72.79%, 67.24%, and 74.69%, respectively. The mean test performance of SCMHBP (71.57%) is close to that of SVM-DPC (72.20%) using the same DPC features. The SCMHBP method is also comparable with the SVM-PSSM400 (75.98%) and SVM-AAindex (76.21%) methods. The score card with best training performance, 86.60%, has a test accuracy of 74.22%, and is adopted in further PCP analysis.

The methods that are presented in Table 4 are compared with SCMHBP. Whereas SCMHBP adopts only a simple weighted-sum classifier and the dipeptide features, the other methods use commonly-used classifiers with only one type of easily-interpretable features. That the combination of complementary features and an ensemble mechanism is widely recognized to improve prediction performance. The ensemble SCMCRYS method for predicting protein crystallization uses propensity scores of *p*-collocated amino acid pairs (*p*=0 for a dipeptide) to the performance of the single SCM classifier uses only dipeptide features [26]. Similarly, the ensemble SVM classifier with multiple feature types is expected to exhibit improved performance.

Consider the methods in Table 4. The SVM and J48 based methods have high training accuracies that exceed 90%. However, the mean test accuracies of the SVM and J48-based methods are less than 80% and 70%, respectively. The J48-based decision tree methods exhibit from an obvious overtraining problem. The performance of the Bayes-based methods is not good. In summary, the SVM-based methods and especially the SVM-PSSM400 method with a mean test accuracy of 75.98% using the normalized PSSM features outperform the J48 and Bayes-based methods.

### Identification of putative HBPs

The use of a scoring function S(P) for predicting a query sequence facilitates the identification of putative HBPs from the putative non-HBPs in which HBPs have not yet been discovered. The unbalanced dataset is reflective of the natural occurrences of HBPs and non-HBPs. The test accuracy, positive and negative predictive values for the SCMHBP method are 74.20%, 0.70% and 99.88%, respectively, on the unbalanced test dataset consisting of 247 HBPs and 90,914 putative non-HBPs (0.27% for HBPs). The positive predictive value is not intrinsic to the test and would be influenced by the putative non-HBPs. To identify potential HBPs from the putative non-HBPs, each sequence P of the 90,914 putative non-HBPs is scored according to the score of S(P). The top-20 sequences are listed in Table 5 including the name, UniProt ID, and annotated function.

**Table 5:** The top-20 putative HBPs according to the HBP sequences.

	Name (UniProt)	UniProt ID	Function	HBP Score
1	Dermatoxin-J2	P86622	Antimicrobial peptide	715.58
2	Antifungal protein	Q08617	This protein inhibits the growth of a variety of fungal species	683.37
3	Dolichyl-diphosphooligosaccharide--protein glycosyltransferase subunit 4C	Q9SF57	May be involved in N-glycosylation through its association with N-oligosaccharyl transferase	673.21
4	Proline, histidine and glycine-rich protein 1	Q8K0G7	Unknown	671.84
5	Eggshell protein 2A	P19469	Unknown	664.84
6	Photosystem II reaction center protein Ycf12	Q0IAK1	A core subunit of photosystem II (PSII)	662.62
7	U13-ctenitoxin-Pn1c	P84018	Acts as a neurotoxin	660.56
8	Histone H3	P83864	Core component of nucleosome	660.43
9	Uncharacterized protein DDB_G0295473	B0G125	Unknown	657.61
10	Vesicle-associated protein	Q06155	May function as a multidomain RNA-binding protein	657.01
11	Uncharacterized protein YML007C-A, mitochondrial	Q3E7A6	Unknown	655.60
12	Sperm protamine P1	P83211	Protamines substitute for histones in the chromatin of sperm during the haploid phase of spermatogenesis.	653.50
13	S-antigen protein	P13821	S antigens are soluble heat-stable proteins present in the sera of some infected individuals.	653.22
14	Uncharacterized 8.4 kDa protein	P08685	Unknown	653.16
15	Glycine-rich cell wall structural protein	P27483	Responsible for plasticity of the cell wall	651.68
16	Defensin-1	P84757	Has antibacterial activity against the Gram-negative bacterium E. coli and the Gram-positive bacteria L. monocytogenes and S. aureus	650.91
17	Putative uncharacterized protein YKL156C-A	Q8TGN0	Unknown	650.84
18	OriE replication initiation protein	D9IEI0	Unknown	650.41
19	Putative uncharacterized protein YEL032C-A	Q8TGP4	Unknown	650.31
20	Protein PCOTH	Q58A44	May be involved in growth and survival of prostate cancer cells through the TAF-Ibeta pathway	648.51

The mean, maximum and minimum scores of sequences in the training dataset HBPGO-TRN1000 are 554.70, 613.90, and 500.70, respectively, when the threshold value is 539.32. All 20 sequences in Table 5 have scores that exceed 648 and so have high potential to be HBPs. Dermotoxin-J2 has the highest score of 715.58 and Protein PCOTH, ranked 20^th^ has a score of 648.51. Interestingly, the eight putative HBPs in Table 5 have unclear protein functions. The high scores of the putative HBPs suggest further experimental confirmation is required.

### Propensity analysis using informative PCPs

Table 6 presents the three physicochemical properties (PCPs) selected by SCM-PCPs. The Pearson correlation coefficients (*R *value) between the PCPs in AAindex and the propensity scores of amino acids help to identify informative PCPs that are useful in further analysis. The three PCPs and their R values are SNEP660103 (R = 0.604), TAKK010101 (R = 0.576) and KARP850101 (R=-0.555). The three PCPs of HBPs are analyzed and discussed below.

**Table 6:** The three physicochemical properties selected by SCM-PCPs.

Amino acid	Heme protein Score (Rank)	^1^SNEP660103 Score (Rank)	^2^TAKK010101 Score (Rank)	^3^KARP850101 Score (Rank)
F-Phe	705.3(1)	0.438(2)	23.0(2)	0.93(19)
H-His	615.6(2)	0.320(4)	11.9(9)	0.982(14)
G-Gly	604.3(3)	-0.073(12)	0.0(20)	1.142(3)
W-Trp	603.1(4)	0.493(1)	24.2(1)	0.925(20)
P-Pro	601.3(5)	-0.016(9)	15.0(7)	1.055(8)
M-Met	599.6(6)	-0.041(10)	11.9(8)	0.947(18)
D-Asp	574.3(7)	-0.285(20)	4.9(14)	1.033(11)
L-Leu	570.1(8)	-0.008(8)	17.0(5)	0.967(15)
K-Lys	568.3(9)	0.049(6)	10.5(10)	1.093(6)
R-Arg	566.1(10)	0.079(5)	7.3(12)	1.038(10)
A-Ala	558.8(11)	-0.110(13)	9.8(11)	1.041(9)
Y-Tyr	541.0(12)	0.381(3)	17.2(4)	0.961(16)
V-Val	535.5(13)	-0.155(16)	15.3(6)	0.982(13)
I-Ile	518.3(14)	0.001(7)	17.2(3)	1.002(12)
Q-Gln	511.2(15)	-0.067(11)	2.4(19)	1.165(2)
T-Thr	498.6(16)	-0.208(18)	6.9(13)	1.073(7)
C-Cys	494.9(17)	-0.184(17)	3.0(17)	0.96(17)
E-Glu	494.6(18)	-0.246(19)	4.4(15)	1.094(5)
N-Asn	468.7(19)	-0.136(14)	3.6(16)	1.117(4)
S-Ser	401.4(20)	-0.153(15)	2.6(18)	1.169(1)

R	1.00	0.60	0.58	-0.56

^1^SNEP660103 = Principal component III.

^2^TAKK010101 = Side-chain contribution to protein stability (kJ/mol).

^3^KARP850101 = Flexibility parameter for no rigid neighbors.

#### A. Aromaticity of the HBP side chains and its contribution

The SNEP660103 property, described as ''Relations between chemical structure and biological activity in peptides'' [33], is ranked 1 by R value (=0.604). SNEP660103 is a composite feature which interprets a set of the PCPs of the 20 natural amino acids as four vectors, extracted via principal component analysis from the ϕ coefficients. The propensity scores of HBPs have the highest *R *value with the vector III, which represents aromatic properties [47]. The large *R *value reveals that the aromatic residues have a higher propensity than the non-aromatic residues to be HBPs.

The aromatic residues with special side chains with aromatic rings and π electrons perform unique functions in proteins such as the hydrophobic interaction, the aromatic-aromatic (π-π) interaction [20,48], and the carrying of mobile electrons in reactions that involve electron transfer [49-51]. Smith *et al*. [20] assessed the chemical composition of the heme binding sites suggesting that aromatic residues supporting the π-π interactions between the residues tend to adopt an off-set, parallel, staggered, or an edge-to-face orientation relative to the heme group. This suggestion reveals the importance of the aromatic residues in maintaining the orientation of heme in HBPs. In investigations of HBP protein activity, the transition metal redox centers in the structures of HBPs are involved in biological electron transfer as mobile electron carriers that shuttle electrons between reductase and oxidase complexes [49-51]. Heme contains an Fe site that can switch between the reduced (FeII) and oxidized (FeIII) states. The molecules that include aromatic π-systems have relatively low redox potentials and a greater probability of undergoing electron transfer reactions. These aromatic residues are thought to increase the redox potentials of heme because the conserved aromatic side chains are close to it. A sequence study showed that the His residue that is located proximally and distally to the heme is highly conserved [52].

The four aromatic residues with the highest SNEP660103 scores are Trp, Phe, Tyr and His [33]. From Table 6 the aromatic residues Phe, His, Trp, and Tyr with scores 705.3, 615.6, 603.1, and 541.0 are ranked 1, 2, 4, and 12, respectively. As presented in Figure 3(c), the two His residues, His39 and His63, are located close to the heme group. These conserved histidines are thought to be involved in the binding of the aromatic donor, forming the catalytic intermediate, and to transfer electrons. The composition of the four aromatic residues (Phe, His, Trp, and Tyr) in HBPs is 12.18% larger than that (10.58%) in non-HBPs (Table 3), supporting SCMHBP with SCM-PCPs, consistent with previous HBP studies.

#### B. The hydrophobic characters of side chain for stabilizing HBPs

The property of TAKK010101 with *R *= 0.576 is described as ''Side-chain contribution to protein stability'' [34]. Hydrophobic interaction influences the stability and various functions of proteins. To estimate the hydrophobic effects, several hydrophobic metrics and various methods of measuring them have been developed. However, a more precise scale that is convenient to use can be obtained by using the free energies that are measured from the difference between the natured and denatured states to quantify the hydrophobic contribution to proteins [34]. This metric quantifies the energetic contribution of the hydrophobic character of each amino acid side chain to the stability of the protein adjusted according to the conformational entropy of the side-chain[53]. The high correlation reveals that the hydrophobic effects have a more important role in the stability of HBPs than of non-HBPs.

Numerous HBPs have a globular structure and hydrophobic bonds have a major role in organizing their self-assembly [54]. HBPs that bind to a heme group become permanently fully folded and stable only upon association with the hydrophobic heme group and the formation of additional hydrophobic bonds [20]. The structures of the holo-HBPs appear to be stabilized by several hydrophobic contacts [55]. These findings are in agreement with the obtained high propensity scores for hydrophobic amino acids Phe, Trp, His and Gly. Notably, a previous investigation found comparably high changes in stability (ΔΔG) for Phe (23.0 kJ/mol), Trp (24.2 kJ/mol) and His (11.9 kJ/mol) [34].

To estimate the contribution of the hydrophobic character of amino acids, the hydrophobic contribution of the side-chain is calculated using the training dataset HBPGO-TRN1000 according to the property TAKK010101. The mean hydrophobic contribution of the side-chain of HBPs is 10.20 kJ/mol, which significantly exceeds that (9.98 kJ/mol) of non-HBPs, according to the Mann-Whitney test with p<0.05. This result reveals that the HBPs prefer hydrophobic residues more than non-HBPs.

Along with the hydrophobic contacts, multiple other interactions mediate the binding of a heme group. These interactions include the formation of coordination bond(s) to an iron ion, van der Waals' contacts between the planar porphyrin ring and protein side chains, and electrostatic interactions that involve the heme propionate substituents and positively charged protein residues [56]. Figures 3(b) and 3(d) present the surface hydrophobicity of 2PBJ and 1CYO. The binding sites of the HBPs (around the heme group) are very hydrophobic. In the presented scoring card, the hydrophobic residues have high scores; these include Phe, Trp, Pro, and Met with scores of 705.3, 603.1, 601.3 and 599.6, respectively. These results demonstrate that the hydrophobic residues have important roles in HBPs. This finding reveals the importance of the hydrophobic interaction between heme and binding sites.

#### C. Inflexibility of HBPs with flexible heme binding residues

The property KARP850101 with *R *= -0.555, indicating a large inverse correlation, ranked 1 using SCM-PCPs is described as ''Flexibility parameter for no rigid neighbors'' [35]. Since the HBPs have various functions and will work across various environments with, for example, various pH values [57,58], the stability of the HBPs is more important. Numerous investigations of HBPs have focused on HBP binding sites and indicate that the binding sites of HBPs are flexible [59,60]. However, those investigations [59,60] did not consider whole protein structures or compare them with those of other non-HBP proteins. Hydrophobic interactions influence the rigidity of protein structures [61]. Accordingly, these hydrophobic residues are postulated not only to compose the hydrophobic binding sites for hemes, but also to maintain the stability of HBPs.

The inverse correlation suggests that the more rigid amino acids have higher scores. The rigidity of a protein affects its structural stability [62]. The fact that the proteins lose their rigidity during unfolding [61] demonstrates that the proteins with less rigidity tend to reach the unfolding state more rapidly. Our results suggest that HBPs are more inflexible for stabilizing HBPs than non-HBPs.

Numerous investigations have found that the binding sites of HBPs are flexible [59,60], but no investigation compared the rigidity of whole HBPs and non-HBPs. To compare the rigidity of HBPs with that of non-HBPs, the B-factor, which is an atomic displacement parameter that quantifies the fluctuation of each atom in the proteins, is utilized to determine the flexibility of proteins [62]. The C_α_ B-factors are extracted from the TargetS311 dataset of HBPs and a dataset of 311 non-HBPs, which provides background values. Table 7 presents the amino acid scores and the mean B-factors of the C_α_ and side chains.

**Table 7:** The amino acid scores and the average B-factors of the C_α_ and side chains

Amino Acid	Score	HBPs	Non-HBPs
F-Phe	705.30	29.41 ± 22.90	28.15 ± 18.41
H-His	615.60	28.38 ± 22.10	30.67 ± 19.56
G-Gly	604.30	32.00 ± 24.25	30.60 ± 20.17
W-Trp	603.10	29.48 ± 22.98	25.18 ± 16.77
P-Pro	601.30	30.60 ± 23.23	32.66 ± 20.87
M-Met	599.60	29.96 ± 22.34	30.20 ± 19.06
D-Asp	574.30	32.00 ± 23.58	34.04 ± 20.96
L-Leu	570.10	31.11 ± 23.27	29.04 ± 18.19
K-Lys	568.30	32.47 ± 22.93	35.76 ± 21.87
R-Arg	566.10	31.83 ± 24.25	33.24 ± 20.55
A-Ala	558.80	29.14 ± 21.42	30.85 ± 20.20
Y-Tyr	541.00	29.73 ± 22.51	26.99 ± 17.72
V-Val	535.50	30.19 ± 22.40	28.02 ± 17.71
I-Ile	518.30	30.53 ± 22.29	29.01 ± 17.89
Q-Gln	511.20	32.20 ± 24.31	33.27 ± 20.80
T-Thr	498.60	31.22 ± 23.47	30.08 ± 19.36
C-Cys	494.90	29.10 ± 23.30	26.61 ± 16.31
E-Glu	494.60	32.62 ± 23.35	35.51 ± 20.95
N-Asn	468.70	31.88 ± 23.42	32.66 ± 20.79
S-Ser	401.40	32.34 ± 23.93	32.13 ± 20.33

R	1.00	-0.45	-0.22
p-value		0.02	0.18

From Table 7 the *R *values between the mean B-factors of residues and the amino acid scores for HBPs and non-HBPs are -0.45 and -0.26 with p-values of 0.02 and 0.18, respectively. The results reveal that HBPs preferentially yield residues with lower B-factors. Although study of the rigidity of HBPs has been published, the results herein suggest that HBPs are less flexible than non-HBPs. Even though HBPs exhibit an allosteric mechanism and these are thought to have flexible binding sites, a statistical study indicates that the allosteric mechanism that is seen in various HBPs, such as the hemoglobin and myoglobin, undergoes a conformational change owing to the side-chain rotamer without backbone changes [63].

**Figure 4:**
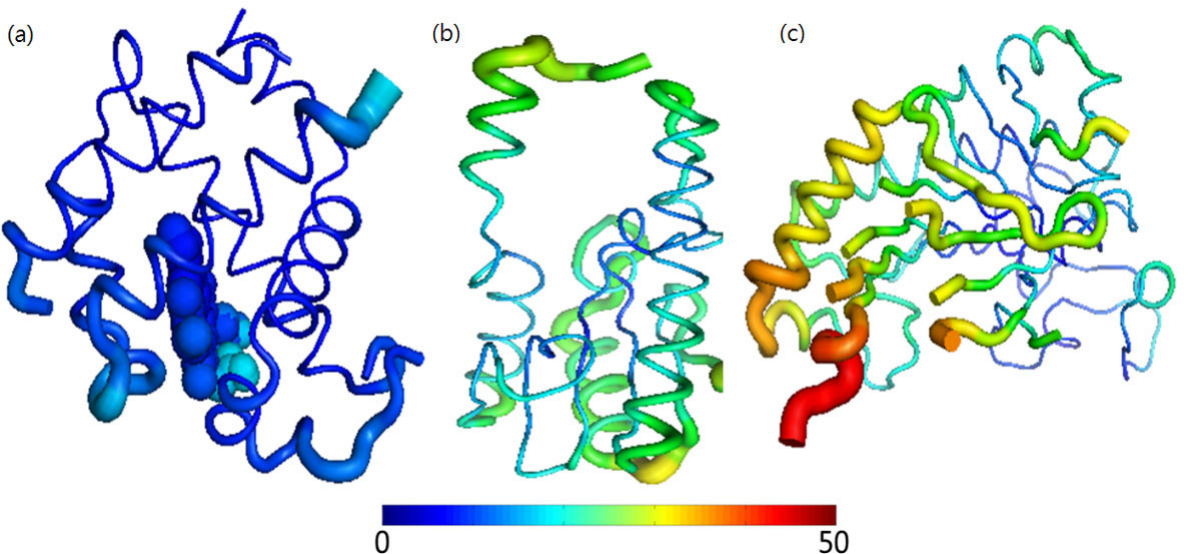
**The B-factor distributions of the HBP (3BL2) and non-HBPs (1CW0 and 1E5H)**. The high B-factors are represented in red while the low value is in blue. The backbones are shown as the traces. In the HBP, the heme group is represented with spheres. The thinner traces mean the lower B-factors both in HBP and non-HBPs. (a) Dehaloperoxidase A. (b) Repair endonuclease. (c) Deacetoxycephalosporin C synthase.

To elucidate the above phenomena, the HBP (3BL2) with the highest score (608) in the Target311 dataset and two non-HBPs (1CW0 and 1E5H) are considered. 1CW0 has the lowest score of 357 and the randomly selected 1E5H has a score of 540. Figure 4 plots the B-factor distributions of the HBP (3BL2) and non-HBPs (1CW0 and 1E5H). The blue residues of the HBP indicate that the B-factor of the whole HBP is low. The green and yellow residues of non-HBPs indicate that the two proteins have higher B-factors than the HBP.

Possible causes of the lower flexibility of the HBPs are as follows. 1) HBPs typically function in various environments; for example, they may transport oxygen to organs or transfer energy in mitochondria. According to a previous circulation study, for example, P_CO2_ affects the pH of the blood [64]. Reducing P_CO2_ increases arterial pH, changing the oxygen binding ability of hemoglobin [57,58]. The hemoglobin must tolerate various pHs of various environments. 2) The HBP is involved in some important signals such as apoptosis [65], which triggers a change in conformation. Domain swapping owing to the protein flexibility of the protein causes domain escape from the original position of the proteins on cytochrome *c_552_*. These domain swapping proteins can further bind to each other to form dimers or oligomers. In cytochrome *c_552_*, if domain swapping occurs, then the protein oligomers will trigger apoptosis.

## Conclusions

This work proposed a scoring card method (SCM) based method (SCMHBP) for predicting and analyzing HBPs from sequences. SCMHBP performs well in terms of mean test accuracy when applied to three independent datasets. Additionally, the propensity scores of dipeptides and amino acids can support the identification of informative physicochemical properties to provide insight into heme binding proteins. The CP motif has high propensity score, suggesting that this motif is important for HBPs. SCM-PCPs are used to identify the physicochemical properties of HBPs and three PCPs, SNEP660103, TAKK010101 and KARP850101, are identified. In summary, the aromaticity and hydrophobicity of the side chains of the HBP residue appear to be important factors in determining the functional performance and stability of HBP. Additionally, HPBs appeared to have a more rigid structure than non-HBPs. The used datasets and source code for SCMHBP are available at http://iclab.life.nctu.edu.tw/SCMHBP/.

## Competing interests

The authors declare that they have no competing interests.

## Authors' contributions

YFL and PC conceived the idea of this work, carried out the system design, and participated in manuscript preparation. YSS and TV conducted the analysis of physicochemical properties and participated in manuscript preparation. PC and SCL implemented the programs. SCL and YHC established the website. HCL participated in the experimental analysis and discussion. HLH and SYH participated in the system design, supervised the whole project and coordination, and helped to write the manuscript. All authors have read and approved the final manuscript.

## Declaration statement

This work was funded by National Science Council of Taiwan under the contract number NSC-103-2221-E-009-117-, and "Center for Bioinformatics Research of Aiming for the Top University Program" of the National Chiao Tung University and Ministry of Education, Taiwan, R.O.C. for the project 103W962. This work was also supported in part by the UST-UCSD International Center of Excellence in Advanced Bioengineering sponsored by the Taiwan National Science Council I-RiCE Program under Grant Number: NSC-102-2911-I-009-101. Publication charges for this work are funded by the project 103W962.

This article has been published as part of *BMC Bioinformatics *Volume 15 Supplement 16, 2014: Thirteenth International Conference on Bioinformatics (InCoB2014): Bioinformatics. The full contents of the supplement are available online at http://www.biomedcentral.com/bmcbioinformatics/supplements/15/S16.
